# An Ecological Approach to Evaluating Collaborative Practice in NSF Sponsored Partnership Projects: The SPARC Model

**DOI:** 10.3389/fpsyg.2021.751660

**Published:** 2022-01-11

**Authors:** Erin M. Burr, Kimberle A. Kelly, Theresa P. Murphrey, Taniya J. Koswatta

**Affiliations:** ^1^Assessment and Evaluation, STEM Workforce Development, Oak Ridge Associated Universities, Oak Ridge, TN, United States; ^2^Independent Researcher, Knoxville, TN, United States; ^3^Department of Agricultural Leadership, Education and Communications, Texas A&M University, College Station, TX, United States

**Keywords:** collaborative practice, assessment and evaluation, higher education, NSF alliance and partnership programs, STEM education and careers, diversity, equity, and inclusion (DEI)

## Abstract

From co-authored publications to sponsored projects involving multiple partner institutions, collaborative practice is an expected part of work in the academy. As evaluators of a National Science Foundation (NSF) Alliances for Graduate Education and the Professoriate (AGEP) grant awarded to four university partners in a large southern state, the authors recognized the increasing value of collaborative practice in the design, implementation, evaluation, and dissemination of findings in the partnership over time. When planning a program among partnering institutions, stakeholders may underestimate the need for, and value of, collaborative practice in facilitating partnership functioning. This method paper outlines an evaluative model to increase the use of collaborative practice in funded academic partnership programs. The model highlights collaborative practice across multiple stakeholder groups in the academic ecology: Sponsors of funded programs (S), Program partners and participants (P), Assessment and evaluation professionals (A), academic researchers (R), and the national and global Community (C). The SPARC model emphasizes evidence-based benefits of collaborative practice across multiple outcome domains. Tools and frameworks for evaluating collaborative practice take a view of optimizing partnership operational performance in achieving stated goals. Collaborative practice can also be an integral element of program activities that support the academic success and scholarly productivity, psychosocial adjustment, and physical and psychological well-being of stakeholders participating in the program. Given the goal of our alliance to promote diversification of the professoriate, the model highlights the use of collaborative practice in supporting stakeholders from groups historically underrepresented in STEM fields across these outcome domains. Using data from a mixed-methods program evaluation of our AGEP alliance over 4 years, the authors provide concrete examples of collaborative practice and their measurement. Results discuss important themes regarding collaborative practice that emerged in each stakeholder group. Authors operationalize the SPARC model with a checklist to assist program stakeholders in designing for and assessing collaborative practice in support of project goals in funded academic partnership projects, emphasizing the contributions of collaborative practice in promoting diversification of the professoriate.

## Introduction

This is a story of model discovery and evolution told from the perspective of the authors, serving on an evaluation team for an Alliance for Graduate Education and the Professoriate (AGEP) partnership grant, sponsored by the National Science Foundation (NSF, 2016). From the inception of the partnership proposal to presently entering the fifth and final year of funding, the evaluation team promoted collaborative practice across stakeholders through focused measurement and reporting. This method paper outlines an evaluative model to assist the stakeholders of similar programs who seek to promote the use of collaborative practice across the academic ecology of a funded program. The model further identifies links between collaborative practice and diversifying the professoriate, the overall goal of the AGEP program, and the theme of this special journal issue.

In March of 2018, program and evaluation partners from a newly funded AGEP alliance (hereafter called “our” alliance) joined partners from all concurrently funded AGEP alliances at the *AGEP National Research Conference* in Berkeley, California (California Alliance, 2018). The purpose of the conference was sharing findings and insights related to increasing the inclusion of groups historically underrepresented in STEM fields at the graduate, postdoctoral, and faculty levels in STEM disciplines, thereby diversifying the national professoriate. Over two days, alliance representatives both contributed to and learned from sessions focused on the conference theme, *Pathways to a Diverse Professoriate.* Nine representatives from our alliance and its predecessor contributed two of 18 plenary talks and three of 29 posters (California Alliance, 2018).

When the university and evaluation partners reflected on the lessons shared at the conference, they identified a common thread woven throughout many of the talks and posters—that of collaborative and connective practice. Systematically pulling this thread in subsequent years revealed the wide applicability of collaborative practice in funded academic partnerships, from proposal design to project implementation, program evaluation, and the dissemination of findings.

In the following sections, the authors outline applications of collaborative practice across multiple stakeholder groups in the academic ecology of funded partnership projects; summarize the range of benefits conferred by collaborative practice on stakeholders; and highlight evidence that links collaborative practice and positive outcomes related to diversity, equity, and inclusion (DEI) in higher education. The subsequent methods and results sections present our alliance as a case study illustrating the use of the evaluative model over the lifecycle of the funded partnership program.

### Collaborative Practice in the Academic Ecology

Collaboration is ubiquitous in human society. When more than one person participates in task completion, the actors (aka stakeholders) must work together in successful ways (aka collaborate). Everyone must participate in collaborative activities as part of life. From an early age, we work together in families, in school, scouts, sport teams, and religious congregations. These collaboration and connection structures are built into our physiology and are fundamental to our psychological identity (Holland, 2020).

Participation in the academy is grounded in collaborative practice, including students and faculty in classes and degree programs, in departments and disciplines, in research and laboratory groups, in mentoring and advising relationships, in campus and community organizations. Contemporary STEM educational frameworks characterize collaboration as a fundamental transdisciplinary skill in education and society (Kelly and Burr, 2019). Partnership and workgroup models span the global workforce in business, industry, government, non-profit, and education sectors. Program sponsors like NSF specifically invest in partnership models like AGEP (NSF, 2016) to achieve national education and workforce goals.

Even though collaboration is a natural part of life, the assumption that collaboration occurs naturally when groups gather may lead partners to minimize the attention it deserves in facilitating partnership function. Effective collaboration does not occur naturally or automatically, it requires intentionality about describing what collaborative practice looks like, how it is implemented, and appropriate outcomes measures. Only in such a context can the benefits of collaborative practice be realized.

As reflected in these examples, stakeholder groups in the academic ecology include: (S)ponsors, whose requirements for partner collaboration and program management drive what (P)artners consider when planning programs, and thus what (A)ssessment and evaluation professionals measure. Findings from program studies form the basis of (R)esearchers’ contributions to the academic literature about collaborative practice and its value proposition in the larger academic and global (C)ommunity. The emphasis on multiple stakeholder groups (SPARC) encourages development of collaborative practice across the academic ecology.

### Range of Benefits of Collaborative Practice

The model emphasizes evidence-based benefits of collaborative practice across multiple outcome domains: project implementation and performance, academic success and scholarly productivity, psychosocial adjustment, and physical and psychological well-being.

**Figure 1:**
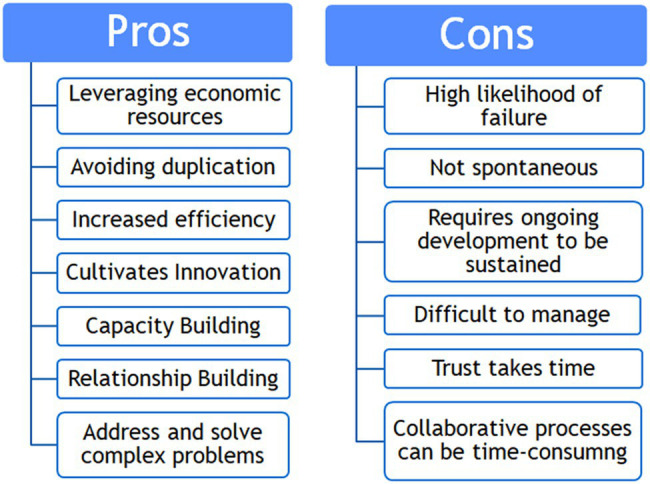
Pros and Cons of Collaborative Practice.

Tools and frameworks for evaluating collaborative practice take a view of optimizing partnership operational performance in achieving stated goals, re-benefits and limitations of collaborative practice in service of project implementation, and performance (Taylor-Powell et al., 1998; Gajda, 2004; Carey et al., 2009; Woodland and Hutton, 2012; Marek et al., 2015). Figure 1 summarizes common pros and cons of working in collaborative partnerships. The benefits (pros) reflect the idea that collaborative partnerships boost program effectiveness by leveraging resources such as relationships, expertise, funding, and unique capabilities across program partners. Partnerships often have further reach with greater impact than partners going it alone. In contrast, the limitations of collaboration center around the challenge and demand of coordination across partners. Any partnership formed must build trusting relationships among the active stakeholders, and this requires extended time spent together. Managing partnerships is difficult and requires considerable sustained effort and interpersonal finesse. Collaborative planning and implementation can be prohibitively time-consuming.

Collaborative practice can also be an integral element of program activities that support the academic success and scholarly productivity, psychosocial adjustment, and physical and psychological well-being of stakeholders participating in the partnership program. Collaborative practice provides important academic benefits “from cradle to career.” Collaboration is part of a transdisciplinary skill set that supports academic and workforce performance over the lifespan (along with communication, critical thinking, and creativity; Kelly and Burr, 2019). Many complex technological and scientific advances require interdisciplinary collaboration and sharing knowledge across diverse disciplines. For example, NSF has committed to investing in their 10 Big Ideas,^1^ which require collaboration across sectors. Research suggests that measurable positive attitudes and behaviors toward cross-disciplinary and interdisciplinary work are related to engagement in collaborative workgroups (Misra et al., 2015).

Academic scholars rely on both formal and informal channels of learning in the academy. The classroom and coursework constitute official pathways for learning requisite disciplinary information for the degree sought. Unofficial channels reflect information learned through interactions with faculty and peers outside formal learning environments. The information learned through such unofficial channels is referred to as the “hidden curriculum” (Elliot et al., 2016). Collaborative practice structures such as mentoring, short-term embedded practice experiences, writing workgroups, and job coaching can provide support that makes this implicit learning explicit. For example, specifically supporting transitions from doctoral to postdoctoral to early career faculty positions through collaborative practice and mentoring addresses this hidden curriculum, as these transitions often lack formal guidance from the academy (Settles, 2020).

Aside from academic domains, collaborative practice supports the psychosocial and sociocultural adjustment of scholars. Ongoing opportunities to collaborate and connect across diverse communities can promote feelings of belonging and inclusion, as time spent together provides the time and space necessary for trust, group identification, and mutual regard to develop (Komives and Wagner, 2017; Micari and Pazos, 2021). Further, a substantial body of research has demonstrated the profound negative consequences that loneliness and isolation can have on the quality and duration of life as well as the mental health and well-being of citizens across the lifespan (Murthy, 2020). Collaborative practice promotes psychosocial connections that can support coping with feelings of isolation and ostracism in the academy and promote scholar persistence (Kelly et al., 2021).

Murthy (2020) clearly demonstrates how psychosocial connection is directly correlated to well-being and life expectancy. Recent research suggests that participation in the academy, particularly in advanced graduate and faculty roles, is significantly stressful and challenging. Advanced degree programs push students’ academic development, but in doing so, they can raise levels of anxiety and depression, particularly near the end of the doctoral program (Bolotnyy et al., 2021). The obvious remedies include connecting scholars with counseling, psychiatric services, support, and recovery groups. Emphasizing activities and discussions about work-life balance, family issues, the pandemic, civil unrest, and wellness habits can provide common experiences among scholars to support their health and resilience (Edwards and Ashkanasy, 2018; Yusuf et al., 2020).

### Collaborative Practice Supports Diversification of the Professoriate

Given the goal of our alliance to promote diversification of the professoriate, the model highlights the use of collaborative practice in supporting stakeholders from groups historically underrepresented in STEM fields across these outcome domains. Diversification of the professoriate and national workforce is a government priority. NSF has operationalized its commitment to diversification in its *Broader Impacts* review criteria used by independent review teams to assess every submitted proposal (NSF, 2021c). AGEP alliances strategically focus on the engagement of doctoral, postdoctoral, and early career scholars who represent groups historically underrepresented in STEM fields.^2^ AGEP alliances promote DEI in both its structure and function. The use of communities of practice as a structure for learning, sharing, and supporting scholars underlies many alliance strategies (NSF, 2021c).

DEI in the academy do not happen naturally. Ensuring that all partners are both represented and participating is fundamental for a successful collaborative partnership seeking to broaden diversity in the academy (Pritchett et al., 2021). Stakeholders may require professional development or expert facilitation to plan and implement effective collaborative practice across diverse stakeholders.

A growing body of evidence links collaborative practice and outcomes related to DEI in higher education. For example, students representing groups historically underrepresented in STEM fields are less likely to possess the connections, networks, or mentoring around them to recognize and encourage them (Yeneabat and Butterfield, 2012; Ponjuán, 2013) or to help them navigate the hidden curriculum (Elliot, 2016; Settles, 2020). Engaging scholars in undergraduate research or other collaborative research settings can help prepare them to enter advanced studies (Jones et al., 2010; Cheruvelil et al., 2014; Hernandez et al., 2018).

Mentioned earlier, ongoing opportunities for scholars to collaborate and connect across diverse communities can nurture psychosocial connections and support health and well-being, both of which influence persistence in the academy. This is particularly important for scholars from groups historically underrepresented in STEM fields, who are at elevated risk in these domains due not only to the difficulty of a higher degree program (Bolotnyy et al., 2021), but also to inescapable systemic racism and ostracism within the academy, and prior experiences in society. These experiences elevate loneliness and social pain, impacting health and well-being. These same students are less likely to seek psychological support services or persist with them (Leong and Kalibatseva, 2011), in part due to potential stigma associated with use of such services.

No paper published in 2020 or 2021 is without a reference to the global pandemic and its major psychosocial, economic, public health, political, and higher education impacts (Usher et al., 2020; Cotula, 2021; Jackson, 2021; Khalil et al., 2021; Lynch and Bambra, 2021). Society changed unexpectedly and profoundly in response to the global pandemic. Social distancing, mask-wearing and stay-at home policies subjected everyone to risk from the trauma of forced isolation from others for an extended period. Research has demonstrated the profound consequences this can have on the health and longevity of citizens across the lifespan (Murthy, 2020). National data further confirm that racial minority groups had higher incidence and hospitalization rates relative to their proportions in the population (Stokes et al., 2020). The pandemic has elevated the health risk of racial minorities more than others.

The literature supports the benefits of collaborative practice across the academic ecology of funded partnership programs. By encouraging a broader conceptualization of the potential benefits of collaborative practice, the proposed evaluative model offers stakeholders from similar partnership programs a tool for considering collaborative practice in their own context. Next, in the methods and materials section, authors provide concrete examples of collaborative practice and their measurement using data from a mixed-methods program evaluation of our AGEP alliance over four years.

## Materials and Methods

The authors served as a program evaluation team, serving primarily as non-participant observers with unique individual positioning. One evaluator came from the lead institution and served as an internal evaluator focused heavily on formative evaluation. The two other evaluators came from the assessment and evaluation group of an external non-profit organization. One external evaluator maintained a primarily administrative and oversight role to ensure evaluation objectivity and contract compliance, while the other external evaluator engaged deeply with the partnership leaders and the internal evaluator to coordinate analysis, reporting, and dissemination of formative and summative evaluation findings. This blended model takes advantage of the increased access to stakeholders by internal evaluators and the requisite need for objectivity satisfied by external evaluators (Patton, 2008).

The lead institution of our AGEP alliance coordinated Institutional Review Board (IRB) approval across the four university partner institutions and the not-for-profit organization of the external evaluation team. Signed informed consent from all program stakeholders (both those receiving programming and those delivering programming) allowed the use of ongoing implementation data collected as part of the project for research and evaluation purposes, such as written reflections, zoom recordings, attendance data, and participant feedback from meetings and events. Specific interview protocols, survey instruments, and other tools such as Individual Development Plans (IDPs) were also submitted for approval, including protocols and instruments used in evaluating collaborative practice. Amendments submitted separately incorporated changes and additional instruments into the original IRB application over the years of the grant.

The program evaluation of our AGEP alliance employed a mixed-method, multi-informant approach to characterize alliance progress in achieving intended outcomes. The evaluation focused on the assessment of collaborative practice across our alliance partners, with stakeholders in the national AGEP community, and in the academic ecology in which they reside.

### Stakeholder Groups of Interest

**Figure 2:**
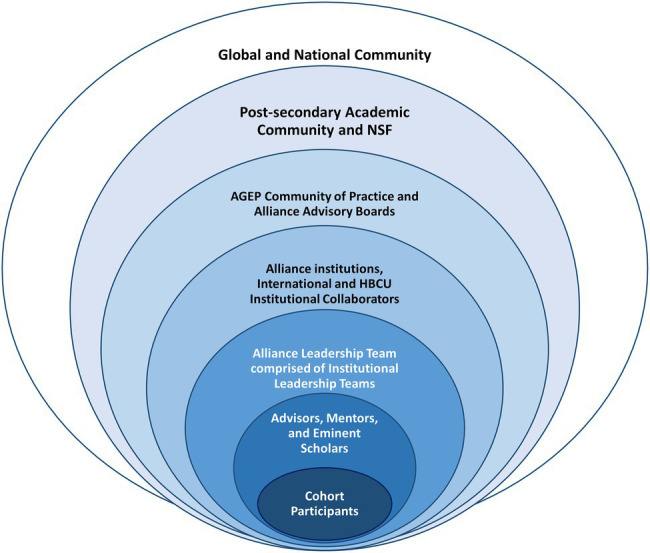
Nested Stakeholder Groups in the AGEP Alliance.

The academic ecology of our alliance, depicted as a set of nested stakeholder groups in Figure 2, reflects the stakeholder groups of concern in the proposed evaluative model. The inner four rings are specific to our alliance, while the three outer rings depict the academic ecology that houses our alliance.

At the core of the model are the cohort participants, the primary targets of alliance programming. Since the emphasis of the alliance was on model development, implementation, and study, the funding sponsor limited cohort size. Nine graduate students from identified groups historically underrepresented in STEM fields recruited across four university partners participated for the duration of the program. Requirements for participation included initial status as a dissertator from a recognized minority group with the intention to seek a postdoctoral or faculty position upon completion of the doctoral program. Several dissertators discontinued their participation in the program in the first year after deciding to pursue work outside of the professoriate. For each cohort participant, the alliance engaged university faculty to serve in three distinct mentoring roles, represented in the second innermost ring.

The third innermost ring contains the leadership team, currently 32 faculty and staff across alliance partners who provide activity programming and partnership coordination. Each participating university partner has a local team that is part of the alliance leadership team, tasked with specific activities or elements of the program model. The evaluation requested that cohort participants and members of the leadership team participate in data collection on an annual basis. Thus, the evaluation employed a longitudinal, census approach that sampled everyone in the populations of interest. Finally, the fourth innermost ring represents the overall institutional context of our five main alliance partners and the supporting international institutions and Historically Black Colleges and Universities (HBCUs) that our alliance has partnered with for specific program activities.

The three outer rings that surround our AGEP alliance represent the academic ecology in which the alliance is embedded. The third outermost ring includes the national community of AGEP alliances and stakeholders of similar programs, representing the research community most proximal to the alliance stakeholders. The AGEP program is located within NSF’s Human Resource Development (HRD) Division of the Education and Human Resources (EHR) Directorate. AGEP’s goal is to “increase the number of historically underrepresented minority faculty in STEM…to fund grants that advance and enhance the systemic factors that support equity and inclusion and, consequently, mitigate the systemic inequities in the academic profession and workplace.”^3^ The community of AGEP alliances connects through annual AGEP national research conferences and other activities relevant to all alliances.

The alliance appointed three advisory boards, one representing stakeholders from the alliance participant cohort, as well as nine subject matter experts from institutions outside of our alliance selected for their research, content, and evaluation expertise in related programs. They provided feedback and professional development to the leadership team and social science research team. The second outermost ring includes the postsecondary education and research academic community at large, with NSF as a major sponsor of research for the STEM disciplines included in this layer. Finally, the outermost ring represents society at large, a reminder that funded programs fulfill national and global needs. In the current context, the need addressed is promoting DEI in the professoriate.

#### Our AGEP Alliance Model

The goal of our interdisciplinary AGEP alliance is to develop, implement and study a model of STEM doctoral degree completion and the transition to successful postdoctoral fellowships and faculty careers for groups historically underrepresented in STEM. A customary way to depict programs like our alliance is with a logic model, a systematically developed visual representation of a program’s underlying assumptions and theoretical framework (W. K. Kellogg Foundation, 2004). Logic models typically delineate the activities of each institutional partner of the alliance (inputs) and connects these activities to their intended outputs (i.e., products of program activities) and outcomes (i.e., specific changes in participants’ behavior, knowledge, skills, status, and level of functioning).

**Figure 3:**
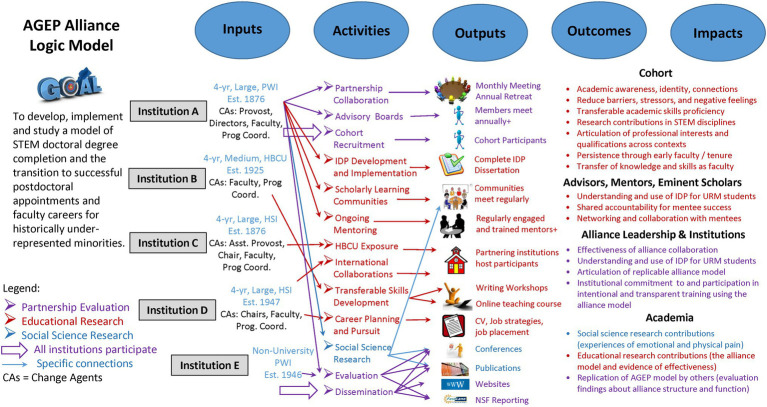
Logic Model for the AGEP Alliance. The logic model displays how the activities of each institutional team lead to outcomes in service of the overall alliance goal.

The evaluation team developed the alliance logic model (see Figure 3) based on program documentation. The logic model maps program elements to three strands of research and evaluation: educational research, social science research, and partnership evaluation. The education research strand is related to the activities offered to stakeholder participants. Local teams responsible for activity development, implementation, and outcomes engage in research to validate observed outputs and outcomes on stakeholder participants. The social science research strand contributes to the larger knowledge base about policies and practices for improving academic outcomes for students representing groups historically underrepresented in STEM fields in higher education. The social science research team examined the relationship between social and physical pain and how this relates to the experiences of students from groups historically underrepresented in STEM fields in the academy.

The evaluation team used the alliance logic model as a basis for designing formative and summative program evaluation. Formative evaluation provides ongoing feedback about alliance functioning in a continuous improvement cycle (during monthly meetings). Summative evaluation focuses on providing credible evidence of program effectiveness in achieving program outcomes (annual reporting). The evaluation strand of the logic model focuses on partnership collaboration, feedback from advisory boards, recruitment and coordinated engagement of cohort participants in program activities, and dissemination across all three research and evaluation strands.

Logic models not only guide evaluation design but are also instrumental in ensuring stakeholders (inputs) specify what they expect to accomplish (activities and outputs) and how they will know if they did so (outcomes and impacts). Ideally, engaging the leadership team in collaborative discussion around the logic model promotes shared understanding of program goals, roles, and responsibilities, and expected outcomes (Kelly and Burr, 2019). The evaluation team traced the development of shared understanding of the alliance model among the members of the leadership team over time and in response to professional development.

#### AGEP Community of Practice (COP)

An export from the public funding portal of NSF (2021a) itemized 27 AGEP alliances since 2013 (18 are currently active). Each alliance identified a lead institution for administrative purposes. In total, 22 different institutions served as leads. Five institutions^4^ have led consecutive or multiple alliances. Each lead partnered with one or more doctoral institutions, ranging from two or three (20% of alliances) to six or more (35% of alliances), with 50% of alliances having four or five partners. As noted previously, a total of five institutions partnered in our alliance, four doctoral granting institutions in a southern state and an evaluation team contracted from a non-profit government organization in another southern state.

**Figure 4:**
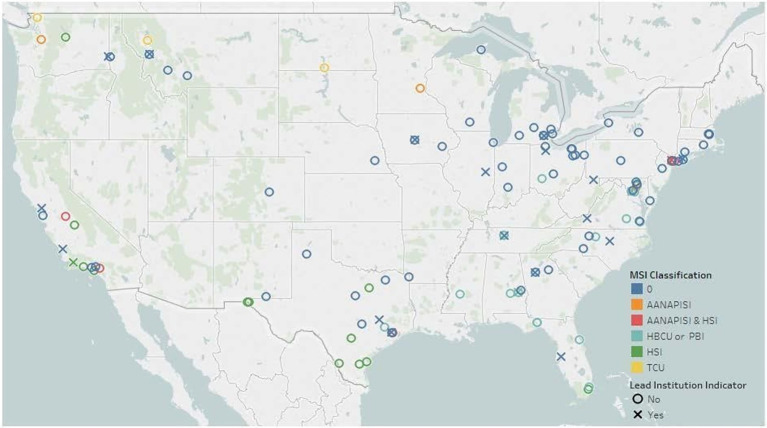
National Map of AGEP Alliance Institutions from 2013-2020. Minority Serving Institutions (MSI Classification) are highlighted in different colors, and the lead institution for each alliance is designated with an X.

Across these 27 alliances, there are a total of 112 unique institutions partnered in one or more alliances. The authors classified each partner using the Basic Carnegie Classification of Institutions of Higher Education (Indiana University Center for Postsecondary Research, 2021) and designations for Minority Serving Institutions (MSIs; U.S. Department of Education, 2020). All institutions are located within the continental United States. On the map in Figure 4, each institution is located as a colored circle representing MSI classification, with lead institutions designated with an ‘**X**’. Of the 112 institutions, 43 (38%) have an MSI designation. Two-thirds of the partnering institutions have doctoral programs with high or very high research activity. The other third includes schools focused on associate’s (*n* = 8), baccalaureate (*n* = 5), and master’s (*n* = 18) degree programs, tribal colleges (*n* = 3), and a few professional doctoral programs (*n* = 4). Figure 3 (inputs column of the logic model) summarizes the characteristics of the four institutions comprising our AGEP alliance.

The AGEP institutional portfolio constitutes the AGEP community of practice (COP). The existence of the AGEP COP provides opportunities for collaboration beyond a single alliance. Further, a steady stream of AGEP-affiliated events provided regular venues in which collaborative practice across alliances encouraged capacity building around common alliance needs. The evaluation team highlighted professional interactions of our alliance members within the AGEP COP.

### Measurement Strategies and Data Sources

The Collaboration Evaluation and Improvement Framework (CEIF; Woodland and Hutton, 2012) informed the program evaluation of collaborative practice in our alliance. The CEIF outlines qualitative and quantitative data collection strategies and measurement tools for each of five entry points to collaborative practice in a partnership:

1. operationalize the construct of collaboration—collaborative structures and strategies
2. identify and map communities of practice—interactions among alliance team members
3. monitor stages of development—assemble/form; storm/order; norm/perform; transform/adjourn
4. assess levels of integration—cooperation (sharing), coordination (co-hosting), collaboration (merging)
5. assess cycles of inquiry—data-driven dialog, decision-making, and action

To describe the collaborative practices employed by or engaged in by alliance stakeholders across the five entry points, the evaluation team relied on three sources of data:

1. ongoing program documentation, annual reporting, and dissemination products
2. observation of alliance events with related attendance and feedback data
3. annual assessment of stakeholder knowledge, attitudes, and behaviors in self-report questionnaires and semi-structured interviews

The evaluation team employed strategies to build rigor into all assessment phases: development, acquisition, and analysis. They worked closely together to develop self-report tools and interview protocols based on the CEIF as well as adapt both the number and details of interview questions and self-report instruments each year as collaborative practice evolved across the leadership team and cohort participants.

Following the utility standard of program evaluations [i.e., attention to stakeholders; Joint Committee on Standards for Educational Evaluation (JCSEE), 2018], we considered all individuals targeted by the project evaluation as the sample of our study. Each year during the spring semester, the evaluation team met with each leadership team member and cohort participants engaged in the funded activities of our alliance. Each year, the evaluation team followed similar procedures for scheduling, reminding, providing copies of the questions in advance, so respondents could complete self-report instruments before the interview. During hour-long interviews conducted on a conference telephone line, one evaluator guided questioning using a semi-structured protocol, while another evaluator scribed detailed notes into an electronic template. This resulted in high quality data acquisition of stakeholder responses. Further, only one or two respondents failed to participate in the data collection request each year, yielding a very high response rate (~95%).

Qualitative analysis involved coding responses to interview questions or other narrative sources of information and unitizing of data (Merriam and Tisdell, 2016). The constant-comparative method (Glaser and Strauss, 1999) entailed comparing data to allow themes to emerge. The engagement of the same evaluation team each year, using the same procedures for coding data and consolidating across respondents, ensured consistency and credibility of the data. Team review of coded data ensured consensus agreement of the final data across the evaluation team. For example, the consistency of answers across respondents and how responses changed over the lifecycle of the project. The next section reviews the interview questions and self-report tools chosen to address each entry point of the CEIF.

#### Self-Report Instruments and Interview Protocols

##### Operationalize the Construct of Collaboration

Interview questions addressed the following topics:

1. shared understanding of the alliance goal and logic model across stakeholders
2. activities and structures for successful collaboration, such as regular meetings, location of shared information and resources
3. plans to address turnover in the leadership team, resolve conflict or disagreements
4. opportunities for face-to-face or virtual interactions for building trust among team members
5. working together to disseminate partnership results or outcomes
6. shared decision-making when developing goals/plans.

##### Identify and Map Communities of Practice

Each year, the evaluation team asked those on the leadership team and in the participant cohort with whom they interacted in a substantive way to identify connections within and across alliance stakeholders using the leadership team, participant cohort, and assigned mentor rosters (fourth year only). Four networking levels classified the number of times individuals were identified as a collaborator. Social network analysis maps created using a social network visualizer (SocNetV-2.4^5^) depict each alliance member as a node at their primary institution and shows connections to those within their institution as well as across institutional boundaries for each year of the partnership. In the fourth year, the evaluation team collected network data in a survey format and included information about the amount of connection time as well as the purpose or content of connections among stakeholders to describe the features of collaborative practice in more detail. The evaluation team requested interviewees to complete the survey in advance of the interview session. While there are multiple metrics of potential use in social network analysis, a detailed treatment is beyond the scope of the model presented here; resources like Taylor et al. (2014) provide a fuller discussion.

##### Monitor Stages of Development

Each year, the program evaluation team selected interview questions aligned to the stages of partnership development as noted below; see Woodland and Hutton (2012) for sample questions.

1. assemble/form—shared clarity around purpose, structures, strategies, leadership
2. storm/order—urgency, resources, turf, expertise, willingness to take on responsibilities
3. norm/perform—implement established and specific activities to accomplish goal
4. transform/adjourn—data related to goals and outcomes to refine, reconfigure, or dissolve the collaboration

##### Assess Levels of Integration

All alliance members rated collaborative practice across alliance partners using the Levels of Integration Rubric (LOIR; Woodland and Hutton, 2012). The LOIR lists five categories of collaboration: communication, leadership, members, decision-making, and resources. For each, alliance partners rate from A to E, with A associated with low cooperation (sharing), to medium coordination (co-hosting) at C, and E associated with high collaboration (merging). Interviewees indicated their rubric-based ratings and discussed their reasons during the interview.

##### Assess Cycles of Inquiry

Ongoing cycles of inquiry include dialog, decision-making, action, and evaluation around a shared purpose based on evidence. The alliance leadership team received feedback about alliance performance from a wide range of sources: formative and summative program evaluation, site visits with NSF staff and AGEP COP experts, advisory board meetings, annual report feedback and partnership negotiations with NSF program officers, and annual alliance-wide meetings. The evaluation team documented how the leadership team responded to and integrated this feedback from the various sources.

#### Document Analysis

The evaluation team reviewed both solicitation and funding documents from the sponsoring organization, NSF. This included the AGEP solicitation, which funded our alliance (NSF, 2016). Exported public funding data defined the project scope, funding, and duration for each alliance (NSF, 2021a). AGEP community announcement emails kept all partnering institutions informed. Core alliance documents included the funded project proposal, logic model, annual reports, and dissemination products. The project director captured all alliance data on a secure drive accessible only by alliance members, and only after they completed human subjects’ certification through CITI.^6^

#### Event Observation

The evaluation team observed meetings, conferences, and professional development sessions both within our alliance and within the AGEP COP. Notes taken by the evaluators or program director from in-person or zoom sessions served as primary data from these events in addition to attendance data. With the increased use of virtual platforms during mandatory stay at home periods associated with the global pandemic, the capture of additional information related to participation in our AGEP alliance annual meetings and workshops became possible. Table 1 lists the types of data captured from virtual interfaces. Virtual events, often recorded and made available after event completion, increased access to event data beyond the original presentation.

**Table 1:** Types of data captured from virtual interfaces.

Virtual data type	Use description
Attendance	Recorded participation by session
Audio/video recording	Captured meeting presentations and discussions
Chat	Captured comments during the live presentations and discussions
Master slide deck	Collected content developed by team members
Online survey software	Collected anonymous pre and post meeting data
Padlet	Collected anonymous responses to open-ended questions on a “wall”

## Results

While the CEIF guided evaluation as discussed in the “Materials and Methods” section, the CEIF focuses on promoting project implementation and performance through successful collaborative practice among partners. The evaluation team recognized a broader range of benefits of collaborative practice at play across the alliance as well as within the surrounding academic ecology, including specific benefits for scholars representing groups historically underrepresented in STEM fields in the academy. To incorporate these additional elements of collaborative practice, the authors articulate an evaluative model for describing the conceptualization and actualization of collaborative practice across stakeholder groups in the academic ecology.

**Figure 5:**
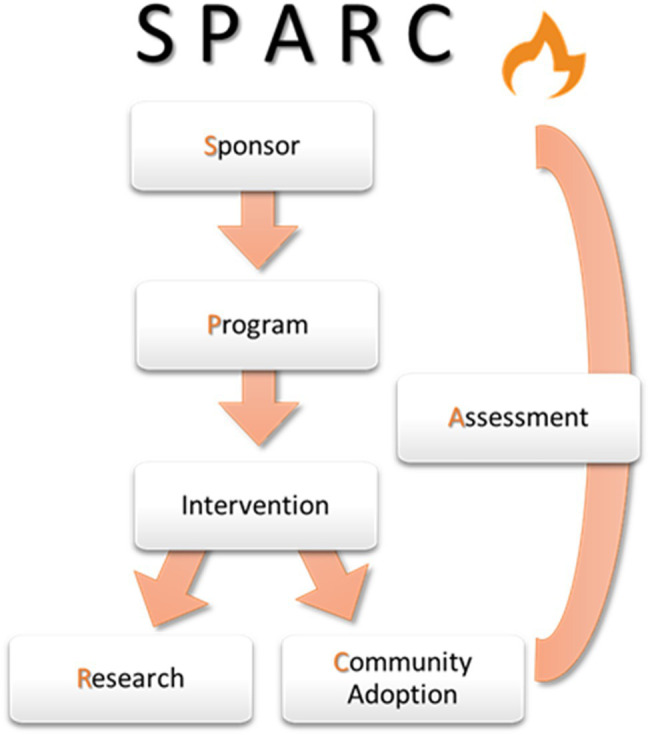
The SPARC Model for Collaborative Practice. SPARC is a framework for examining how stakeholder groups in the academic ecology conceptualize and actualize collaboration structures and processes in strategic partnerships.

Dubbed the SPARC model, this acronym emphasizes collaborative practice across the academic ecology of an educational partnership program and demonstrates the unique contributions of each stakeholder group. Shown in Figure 5, (S)ponsor requirements for partner collaboration and program management drive what (P)artners consider when planning programs, and thus what (A)ssessment and evaluation professionals measure. Findings from program studies form the basis of (R)esearchers’ contributions to the academic literature about collaborative practice and its value proposition in the larger academic and global (C)ommunity. The SPARC model encourages a broader conceptualization of the potential benefits of collaborative practice for stakeholders across multiple outcome domains: project implementation and performance, academic success and scholarly productivity, psychosocial adjustment, and physical and psychological well-being. Of particular emphasis are specific benefits for scholars representing groups historically underrepresented in STEM fields in the academy.

Grounding the evaluation findings in the SPARC model allows a systematic discussion of the role responsibilities of each stakeholder group in the academic ecology, and how they engage in or facilitate collaborative practice. Representative data organized around key analytic themes provide examples of the benefits of collaborative practice in support of alliance and stakeholder success. Each theme summarizes supporting evidence from our alliance program evaluation, detailing the data sources, measurement strategies, and analytic interpretations for each theme. The intention is to illustrate the types of data and insights about collaborative practice resulting from use of the SPARC model rather than attempt a comprehensive presentation of collected evaluation data. A final consideration to keep in mind when reviewing the results is that the evaluation team and evaluation plan evolved over the lifecycle of the partnership as did our leadership team and alliance model, and is still a work in progress.

### Sponsoring Organization (S)

Program officers at the sponsoring organization:

1. specify the details of the solicitation
2. sponsor independent peer review of submitted proposals
3. negotiate the project specifications for award in the form of a cooperative agreement
4. conduct site visits
5. support annual meetings and collaborative opportunities for all award recipients
6. review and approve annual reports
7. release funding increments on behalf of the sponsoring organization

The sponsors of a program influence its structure and function from conception to completion. Sponsor representatives prepare specific funding requests in alignment with policy, plans, and funding allocations, thereby actualizing collaboration requirements for project partners. Proposal review, award negotiations, and reporting requirements for grantees further shape the design and implementation of collaborative practice in funded projects. NSF outlines its policies for sponsored projects in a regularly updated guide to grants (NSF, 2021c).

In proposing partnership projects in response to an NSF program solicitation, the evaluation team examined how program officers or sponsor representatives communicated collaboration requirements or preferences to program partners. Specifically, authors documented the communication of collaboration requirements in the AGEP program solicitations, in award negotiations of our AGEP alliance with the NSF program officer, and in ongoing feedback processes like annual reporting and site visits. Section “AGEP Community of Practice” highlights numerous ways NSF program officers regularly engage the AGEP COP in collaborative opportunities such as proposal review, site visit teams, and conference hosting and attendance.

#### Solicitation Requirements

The evaluation team carefully reviewed the AGEP solicitation (NSF, 2016), which funds our AGEP alliance, for language concerning collaboration and coordination vs. independent activities (Kelly et al., 2020a,b). The solicitation analysis revealed:

Required/Suggested Elements

1. *Partnership requirement.* Must include project partners
2. *Evaluation of collaboration.* Suggests evaluation resources to evaluate collaboration (Korn, 2008)
3. *Definition of Partner Roles*. Define the roles of each partner
4. *Value-Add of Partners to Collaboration*. Prompts for a discussion “why each partnering institution/organization has been selected” as well as “benefits” or “collaborative” contributions
5. *Resources Allocated to Collaboration.* Explicit plan and budget to manage the collaborative aspects of the program
6. *Dissemination to Research Community.* Explicit plan for dissemination of work to the research community

Not Required or Elaborated

1. *Collaboration plan requirement.* Formal collaboration plan
2. *Evaluation of collaboration.* Explicit evaluation of collaborative efforts
3. *Structures for Regular Collaboration/Communication.* Discuss role of collaboration in alliance success or elaborate on structures to use

Analysis of the AGEP solicitation revealed a lack of specificity about articulating collaborative practice at the proposal stage. The requirements do include an explicit plan and budget to manage program collaboration. However, the requirements do not require formal evaluation of collaborative practice or a formal collaboration plan. A potential alliance might not think about the mechanisms of actual collaboration beyond identifying who does what and how the budget supports these roles. Sponsors of such programs should carefully consider how much detail to require in solicitation documents, as the formal requirements will influence how carefully partners plan aspects of the proposed alliance.

#### Award Negotiation and Annual Continuation

During the funding negotiations, the program officer emphasized collaborative practice in several ways, beginning with creating an explicit alliance structure for equitable engagement across partners. As a result, each institutional partner submitted a collaborative research proposal to lead specific elements of the alliance. Further, each partner appointed a coordinator for their institution to support the alliance while the lead institution appointed an overall alliance director.

An AGEP program officer directed the external evaluation team to prioritize collaborative practice in the evaluation over effectiveness of individual intervention elements. The program officer also suggested an internal evaluator from the lead institution as a member of the leadership team, and that faculty with evaluation expertise serve on the advisory board. Finally, the program officer supported using the American Evaluation Association^7^ as a source for relevant expertise. The evaluation team recruited both evaluation experts through their association with AEA. The external evaluators actively participate in AEA and serve leadership roles in the STEM Education and Training Topical Interest Group (TIG).^8^ This involvement allowed the external evaluators to quickly locate appropriate evaluation expertise for our alliance.

Ongoing approval of alliance funding was dependent on submitting annual reporting documents as well as participating in site visits guided by NSF staff. For example, in response to a site visit held in year two of our alliance, supplemental support provided for face-to-face annual meetings improved the quality of alliance engagement and collaboration among alliance stakeholders. Increased funding also supported participation of the evaluation team in AGEP COP programming, along with a specific COP dedicated to evaluation capacity building. From our experience as evaluators, the program officers of the AGEP program have directly and deeply engaged with the partners of all 27 alliances that have been funded since 2013. All these actions during the negotiation and continuation discussions represent significant support of collaborative and equitable practice by the program sponsor.

### Partners and Participants (P)

Program partners and participants:

1. recruit program partners
2. design, prepare and submit a detailed proposal to the sponsoring organization, including elements related to collaborative practice
3. implement the program with participants recruited from partner institutions
4. participate in AGEP COP activities (such as an annual research conference)
5. study and disseminate findings to NSF in an annual report
6. submit presentations and publications to the larger academic community

Program partners plan and implement collaborative practice as part of a funded program, guided both by sponsor requirements and supported by credible research. Planning begins at the proposal phase with the selection of institutional partners and the proposal preparation process used to design the partnership program. One way to infer the value project partners placed on collaborative practice was inclusion in proposal documents and project models. Upon funding, the focus on collaborative practice shifts to how the alliance leadership team works together to launch the partnership, recruit the participant cohort, and implement planned activities of the alliance model over time.

Not only is collaborative practice used by the alliance leadership team to implement partnership activities, once the leadership team recruits the participant cohort, they become actively involved in collaborative practice as part of their alliance participation as scholars from groups historically underrepresented in STEM fields in the academy. The program evaluation focused not only on how collaborative practice improved partnership performance in implementing the model, but also how it promoted academic success and scholarly productivity, psychosocial adjustment, and physical and psychological well-being in the participant cohort.

While the evaluation team examined the role of collaborative practice over the lifecycle of our alliance across all stakeholders, the following two sections will focus on collaborative practice findings relevant to our alliance leadership team during proposal, launch, recruitment, and project implementation phases of the alliance. The implementation discussion also highlights academic and psychosocial benefits of collaborative alliance activities identified by cohort participants. The authors consider this to be one of the most important findings of our alliance evaluation to date.

#### Collaborative Planning

The alliance team leveraged several collaborative strategies in developing our alliance AGEP proposal. Foremost, the alliance team built our alliance upon an existing AGEP partnership, proposing a new AGEP alliance model for implementation in the same university system. The four university partners came from the prior alliance, as did most of the cohort participants. Selection of the external evaluation team by the AGEP alliance occurred as a direct result of collaborative work in another NSF partnership community, the National Research Traineeship (NRT) program.^9^ Representatives from NRT partnerships engaged in a cross-partnership interactive planning activity during an NRT Evaluator’s Workshop, which eventually led to the authors joining our AGEP alliance as external evaluators. Evaluators were involved from the initiation of the proposal process, ideal for proper alignment of program and evaluation design (Kelly and Burr, 2019). In these examples, preexisting collaborative connections facilitated the formation of the current alliance.

Facilitated collaborative grant planning and writing commenced several months preceding the proposal deadline. Professional facilitators appointed by the lead institution guided the leadership team in proposal development. With a large leadership team, this was an important aspect of the proposal process. Consultants who can facilitate a collaborative grant-writing process are an asset to any partnership project. Research Development offices are often useful resources for this expertise. There are also tools and protocols designed to facilitate this process. The National Organization of Research and Development Professionals provides information about these types of resources.^10^

#### Collaborative Implementation

Once funded, our alliance undertook the difficult yet transformative work of evolving collaborative practice across all alliance stakeholders. Using the five entry points of the CEIF framework to explore collaborative practice in our alliance for evaluation purposes, the next section “Assessment and Evaluation Professionals (A)” on assessment and evaluation summarizes evidence of the evolution of collaborative practice across the alliance leadership team to facilitate partner equity, improve cohort engagement, and increase the breadth of program dissemination.

In thinking about other benefits of collaborative practice beyond improving partnership performance in meeting stated goals, one event during the third year of our alliance created opportunities to recognize and document benefits of collaborative practice on academic, psychosocial, and well-being outcomes. This event was none other than the coronavirus pandemic that stopped the world in its tracks with citizens quarantined in their homes early in 2020.

The entire AGEP community had to consider changes in program implementation due to national and international restrictions on movement outside the home. Because most alliances have partners separated geographically, virtual technology was already a part of most alliance operations, including ours. Our AGEP alliance adjusted most programming to a purely virtual environment and managed the impact on the grant budget in response to the pandemic. Activities that engaged cohort participants in place-based professional development experiences were most impacted by the restrictions of coronavirus on travel, including institutional visits to international and HBCU destinations. While most work was and continues remotely, it is not possible to fully replace the place-based experiences planned for these activities. The local institutional teams are planning to complete implementation on a delayed timeline.

Considering the importance of face-to-face activities in the development of collaborative groups, the leadership team was particularly concerned about having to conduct the annual all-alliance meeting planned for June 2020 using the Zoom platform. The leadership team understood the importance of bringing all alliance partners together and made deliberate efforts to make the virtual experience engaging and meaningful. The engagement in the virtual space was successful—the emotional reaction to the meeting was palpable in the faces, voices, and chat comments of the participants.

The evaluation team took advantage of data provided by the virtual platform to describe what happened (Table 1). Table 2 summarizes attendance and chat narrative that supports the successful engagement of alliance stakeholders. The average number of chats each cohort participant received from attendees about their individual presentations provided direct evidence of the affirmation of cohort participants during the virtual meeting. Some of these messages included offers to connect cohort members to career resources.

**Table 2:** Participation results from virtual annual meeting.

Role Group	Attendees		Participated in chat		Total chats submitted	
	*n*	%	*n*	%	*n*	%
AGEP cohort	9	100	9	100	141	27
PI/Co-PI	15	100	12	80	94	18
Senior personnel	4	100	4	100	63	12
Support staff	5	100	5	100	62	12
Evaluator	2	100	2	100	27	5
Graduate assistant	2	67	1	50	1	1
Postdoc	1	100	1	100	20	4
Advisor/mentor	12	71	9	75	69	13
Alliance Advisory Board	6	100	4	67	22	4
Social Science Advisory Board	4	80	4	100	14	3
NSF Program Officer	1	100	1	100	3	1
Total	61	93	52	85	516	100

Stated outcomes for alliance cohort participants on the logic model (Figure 3) include the reduction of barriers, stressors and negative feelings as well as fostering academic identity and connections. Through active engagement in a cohort configuration, alliance participants had opportunities to develop relationships, trust, and a COP among their cohort peers while participating in workshops focused on academic skills development. Regular scholarly learning community (SLC) meetings facilitated ongoing connections among participants and with leadership team faculty during the height of the pandemic. Cohort participants indicated that they continued their own COP outside the alliance (Kelly et al., 2021), and that informal interactions outside of the project were most impactful in building trust and forming bonds. Cohort participants claimed the connections among their cohort peers were essential for their persistence in the academy. The mutual respect, pride, and affection among cohort members provides meaningful and substantive psychosocial support, which promotes both wellness and academic persistence among cohort participants.

### Assessment and Evaluation Professionals (A)

The evaluation team:

1. assists in the design of the program during the proposal phase
2. provides expertise in logic and program modeling
3. develops survey and assessment instruments
4. offers experience in human subjects’ protections
5. designs formative and summative evaluation plans
6. implements the program evaluation
7. provides formative feedback at monthly leadership meetings
8. provides summative feedback in an annual evaluation report
9. disseminates findings in presentations and publications to the AGEP and academic communities.

As the AEPs for our alliance, the authors chose to make the evolution of collaborative practice the primary focus of annual program evaluation. This was also a recommendation of the NSF program officer during grant negotiations. By highlighting the value of collaborative practice in evaluation findings and recommending actions to improve collaboration practice among stakeholders, AEPs encourage attention to the evolution of collaborative practice across the academic ecology. Findings in the following sections reflect the five entry points of the CEIF (Woodland and Hutton, 2012, summarized in section “Self-report Instruments and Interview Protocols”), and include defining each entry point, identifying key constructs and measurement strategies, and summarizing supporting data drawn from our AGEP alliance.

#### Operationalize the Construct of Collaboration

Operationalizing collaborative practice refers to identifying collaboration structures and strategies to guide partnership functioning. There is a need to identify what collaborative practice looks like in the context of our AGEP alliance, creating a shared understanding across stakeholder groups. This is related to the need for intentionality in developing an effective partnership discussed in the introduction. Recall that literature supports improving partnership functioning through collaborative practice.

The size of our overall alliance leadership team required explicit attention to coordination and communication strategies, the underpinnings of collaborative practice. Further, the varied sizes of local institutional teams motivated the leadership team to develop additional strategies to ensure the equitable participation of all partners in decision-making and input into administrative alliance discussions. In the first two years of funding, the alliance leadership team applied feedback from evaluators, the advisory board, and during NSF site visits to improve alliance coordination and communication in service of program implementation.

Meeting protocols used the Zoom platform, recorded for asynchronous viewing. Local institutional team meetings typically occurred the week before monthly leadership team meetings engaging all partners in collaborative planning and discussion. Structures to facilitate effective meetings included attendance and roll call strategies to ensure partner input during decision-making discussions, bounding meeting discussions in time with standardized agendas, and providing minutes and materials from each meeting to all attendees.

Each institution designated a project coordinator to facilitate collaborative practice on behalf of the institutional partner. The lead institution appointed the alliance director, who served as the coordination point for alliance operations. A single point of contact for the overall alliance as well as for each partner institution ensured a high degree of coordination. The director launched the use of project management software (Trello), centralized file sharing (dedicated partnership Google drive), and centralized record keeping (master spreadsheet to track activity delivery and attendance).

In annual interviews, leadership team members acknowledged increased alliance coordination over time because of these actions. While all these strategies were helpful, differing levels of experience and comfort with selected technologies across the leadership team resulted in incomplete adoption. While they understood their importance, many leadership team members noted feeling inundated at times with the constant flow of emails and details from the director. These issues are difficult to balance entirely across such a large team. In all, the leadership team made concerted efforts over time to improve collaborative practice across a large team through the strategic coordination of information.

#### Map Communities of Practice

Mapping communities of practice entails tracking interactions among alliance stakeholders. The activities of our alliance occurred through a network of collaboration. Indeed, the work of most partnership projects occurs at the level of interacting stakeholders across a network of stakeholders. Thus, these connections represent the implementation of the alliance across the academic ecology. Social network analysis and mapping tools effectively model these collaborative networks.

**Figure 6:**
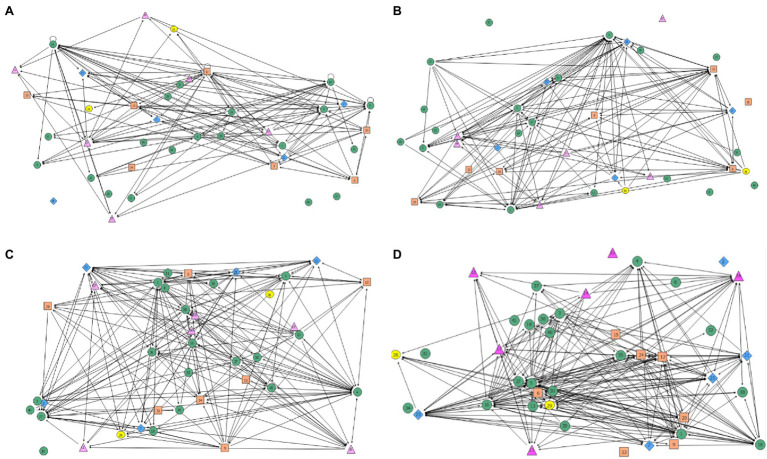
Annual Collaboration Network Diagrams Across AGEP Alliance Institutional Leadership Teams. Each color/shape represents an institutional partner, with individual members designated by random numbers. Panels **(A–D)** show the evolution of connections across the four years of the alliance.

The evaluation team asked each interviewee to identify those with whom they interacted in a substantive way during each year of our alliance. Using these data, analysis examined levels of connection (how many times each alliance member was identified as a collaborator). Connection maps, which represented who is collaborating by connecting two nodes (persons of partnering institutions) in the network with a line, model these connections across all partners (see Figure 6).

Four networking levels represented the number of times each partner was named as a collaborator: Very High (being identified 10 or more times), High (six to nine times), Low (four or five times), and Very Low (three times or less). The alliance PI and the alliance director were identified as Very High each year (essential connections). Partners identified as High were activity leads and coordinators who typically collaborated with those on their campus and with a few others across institutions. Individuals identified as Low or Very Low in connections tended to be those who were new to the project or worked primarily within their institution, with fewer connections outside their local team.

Over four years, eight additional leadership team members were identified as Very High. While only one campus had Very High partners in the first year, three campuses had Very High partners in the second and third years of the program, and all four institutional partners had Very High representatives by the fourth year. While three of the four institutions gradually increased networking over the course of the project, one institution showed decreased networking. Interview comments corroborated the network data, as members of the institutional team expressed feeling disconnected from decision-making and activity implementation. In another case, increasing collaboration with partners across institutional teams compensated for the lack of connection experienced with members of the local team. This also promoted increased alignment of alliance activities that provided complementary benefits (job search and preparation activities aligned to skills development activities).

Social network analysis helped identify patterns of collaboration among members of the leadership team over the duration of the grant. The network maps in Figure 6 illustrate the density of the network connections among team members each grant year. It depicts connections both within and across institutional boundaries. Immediately, it is easy to see that the density of network connections increases over time. Using this network data, the degree of centrality calculation is conceptually like levels of engagement. Over time, centrality spread from one or two members in the first two years to several members by the fourth year. At the beginning of the project, most of the contacts were from the alliance director toward the leadership team members across partnering institutions. From the second year onward, the alliance director becomes the heart of the network (higher degree of centrality). The national evaluation of the NSF AGEP program emphasized the importance of having project directors for alliance stability (American Institutes for Research, 2011).

In particular, the cohort participants indicated how important their relationship with the alliance director was in their project engagement and expressed distress at the turnover in the position in the third and fourth years of the alliance. The turnover of the alliance director role affected participants’ experience of project continuity and commitment, and members of the leadership team expressed similar sentiments during interviews.

The centrality analysis also indicated that internal and external evaluators increased their centrality over the years, with alliance members seeing evaluators more as team members over time. The overall point is that network analysis provides valuable information about how individual partners collaborate. This is useful both for confirmatory analysis as well as a design tool to look for places to encourage or strengthen connections and monitor network growth in response to programming decisions.

#### Monitor Stages of Development

As reviewed in section “Self-report Instruments and Interview Protocols,” Woodland and Hutton (2012) noted that collaborative teams follow predictable stages of development including: assemble/form, storm/order, norm/perform, and transform/adjourn. The CEIF provides a set of questions addressing the pertinent issues that arise during each stage of development, reflecting the typical progression of a partnership’s function over its lifecycle. Questions are both repeated and replaced over time, providing information about developmental changes in partnership functioning. These questions also serve as an important reminder that partnerships should expect to progress through stages of development, each with its setbacks and victories. The progression of our alliance through these stages benchmarks the development of collaborative practice over the lifecycle of the grant.

Proposals identify preliminary levels of collaboration for activities associated with the assemble/form and storm/order stages of development. The assemble/form stage occurred during the first year and the early part of the second year of the funded alliance. As in the discussion about operationalizing collaborative practice, the assemble/form stage of development includes building shared understanding around goals, enacting governance structures, strategies, and leadership.

The program evaluation report articulated the need for shared understanding of the goal of the partnership project and how to conceptualize the alliance model, and a site visit panel provided similar feedback. Shared understanding of the project goal and the alliance model improved over the lifespan of the alliance through alliance wide discussions of stakeholder feedback, with the leadership team members making the shift from an intervention-focused model to an alliance wide partnership model. Answers to annually repeated interview questions about partnership progress toward goals served as data. The similarity of experiences negotiating understanding of the model vs. the intervention shared by many alliance teams suggests this shift is a common event in the developmental trajectory of an AGEP alliance.

The storm/order stage occurred during the first year and continued during the second year of the funded alliance. During this stage, the alliance moves forward with a shared vision, and the business of preparing for activity implementation begins. Storm/order is a descriptive name for this stage, reflecting the often urgent and sometimes chaotic processes of coordinating timelines for the range of alliance activities and providing a coherent plan for the cohort participants to anticipate. At first, coordination was lacking and cohort participants requested more proactive timelines. Planning for data collection needs from cohort participants lacked coordination across institutional teams. Institutional teams collected information for planning purposes from the participant cohort separately rather than employing a centralized strategy that better controlled the burden on participants. This approach left both the leadership team and the cohort participants with a disconnected view of the overall alliance model. Part of the reason for this disconnection was the partnership structure. A consequence of increasing the equitable engagement of all partners in the alliance model through institution specific roles was a siloing effect, limiting information transfer across activities and increasing the difficulty of alliance coordination across activities.

As practice makes perfect, so did time on task improve coordination among alliance partners. After the ordering phase of a partnership, members proceed to the norm/perform stage. The norm/perform stage began toward the end of the second year and continued through the third and fourth years of the funded alliance. The primary focus is the implementation of planned activities to accomplish outcomes in service of our alliance goal, considered the main operational phase of the funded partnership. During the third year of the alliance, the, the coronavirus pandemic disrupted global operations. As previously discussed in Collaborative Implementation, all alliances had to immediately reassess their implementation plans and associated budget allocations.

Our alliance demonstrated an ability to adjust programming and still provide high quality experiences to alliance stakeholders, such as a highly successful virtual annual meeting. While our alliance completed most planned activities despite the limitations imposed by the pandemic, all the stakeholders remain engaged in completing the remaining activities, including those displaced due to the pandemic. As our alliance enters its fifth and final year, implementation of the project continues, shifting over time as some activities conclude and cohort participants transition into postgraduate and early career faculty roles. The content of alliance activities shifts as well to address the concerns of cohort participants in postdoctoral and faculty roles rather than as dissertators.

The transform/adjourn stage began in the fourth year and is continuing into the fifth and final year of our funded alliance. This stage, referred to as transform/adjourn, reflects the transition of the primary focus of the partnership from project implementation to reporting, dissemination, and sustainability. While our alliance has engaged in dissemination activities throughout our alliance lifecycle, it is of particular focus toward the end of a partnership. Given the purpose of federal funding agencies to share and replicate best practices, our AGEP alliance developed a formal dissemination plan. This plan involves a constellation of venues, from peer-reviewed journals to conferences and communities of practice in research, education, evaluation, and broadening participation.

Our alliance is currently developing web pages to showcase our scholarly contributions to a public readership. Leadership team members are also developing a virtual toolkit to share best practices based on our alliance model more broadly. As a result of reliance on the virtual mode of content delivery during the coronavirus pandemic, our alliance utilized a range of virtual tools to increase engagement and enhance program delivery on digital conferencing platforms like Zoom. The toolkit will showcase this repertoire of virtual tools. While the pandemic profoundly disrupted global society and higher education, it also provided a space for new knowledge to arise, and the emphasis on virtual technology as a tool to combat isolation and oppression is one example.

#### Assess Levels of Integration

The CEIF suggests an important feature of a partnership is the integration of activities across partners (Woodland and Hutton, 2012). Integration exists as a continuum that ranges from lower to higher levels of integration. At the lower end, partners simply share information or resources in a cooperative fashion. In the middle, partners coordinate more closely to accomplish the goals of the partnership, a co-hosting arrangement. At the high end, collaboration requires an effortful, yet beneficial, merging of mission, materials, and processes. An important clarification is that optimal levels of integration will depend upon the needs of the partnership, and integration may vary across functional domains.

Each year, the evaluation team used the LOIR to assess and describe the functioning of the strategic partnership. The levels of integration range from cooperation (sharing) to coordination (co-hosting) to collaboration (merging) using a grading scale of A (lowest) to E (highest). Each leadership team rated integration each year across five functional domains: communication, leadership, members, decision-making, and resources.

**Figure 7:**
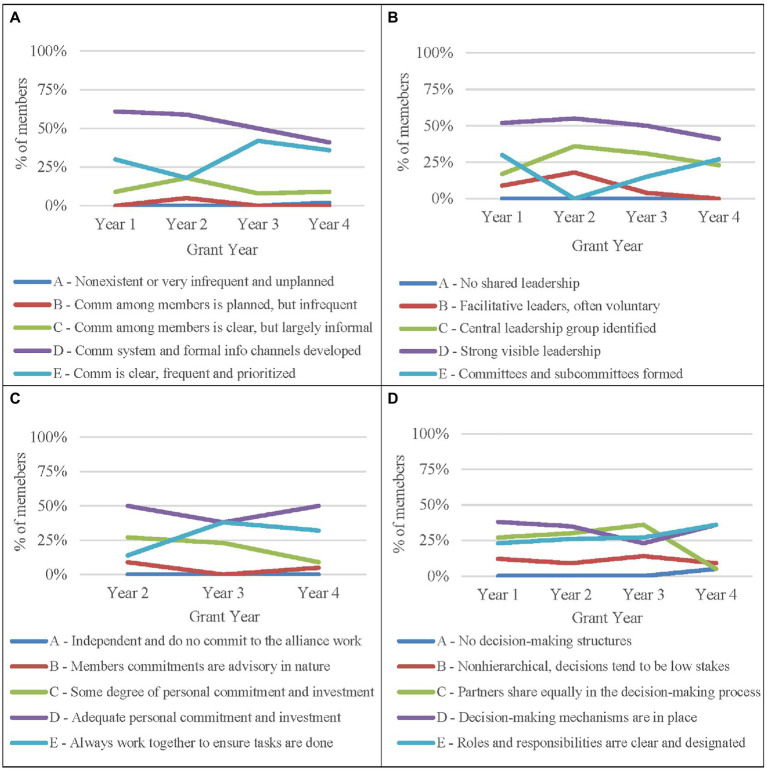
Annual Levels of Organizational Integration for Communication, Leadership, Members, and Decision-Making.

Figure 7 illustrates findings from four of the five domains across four years of the funded alliance. Alliance members rarely selected rubric scores of A and B, indicating that for collaborative constructs under consideration, alliance members established relationships that went beyond simply sharing to co-hosting and collaboration, reflecting more integrated partnering. Ratings in Figure 7D show that ratings of decision-making varied over the first three years, but converged to ratings D and E in the fourth year, reflecting a more consistent perception of collaboration.

Regarding the resources domain, recall that grant negotiations with the program officer resulted in independent budgets allocated to each institution based on assigned activities. This reflects a sharing arrangement, which is at the lower end of the integration rubric. Leadership team members consistently reported difficulty in applying the rubric to the resources domain, and many chose not to answer because integration did not seem to apply as the budgets were independent. Taken in sum, data from the LOIR reflected changes in the perceptions of integration over time and domain in response to programmatic decisions and progress.

#### Assess Cycles of Inquiry

The final CEIF entry point, assessing cycles of inquiry, focuses on how partners engage in data-driven dialog, decision-making, and action. As partnerships proceed through stages of development, how do stakeholders negotiate change? Change is expected and important in a partnership project. NSF has a section in annual reporting that specifically addresses changes in scope, budget, or implementation that occur during the lifecycle of a funded project.^11^

**Figure 8:**
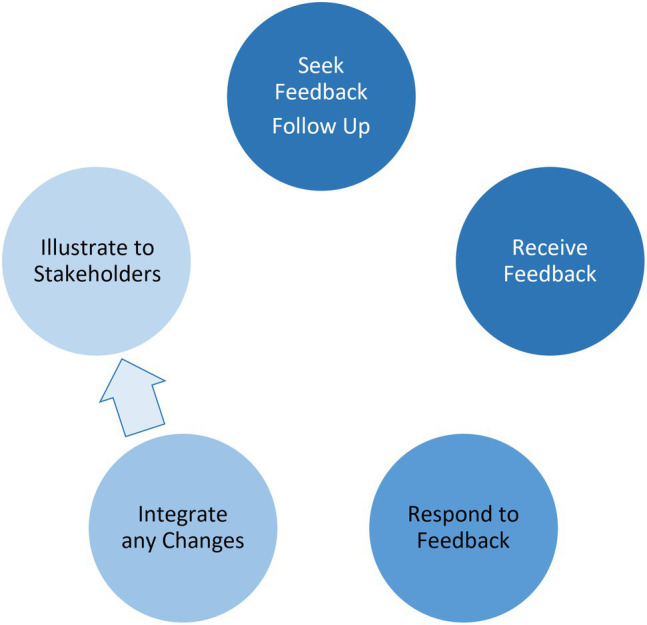
Feedback Response Cycle for Managing Change in the AGEP Alliance. The lighter circles illustrate incomplete attention to bringing evidence of change back to stakeholders.

Using a feedback response cycle (Figure 8), the evaluation team examined how the alliance leadership team engaged in seeking feedback and implementing changes in alliance function. Sources of feedback, or inputs into the feedback cycle, were numerous. These inputs included annual evaluation and reporting requirements, alliance annual meetings, site visits and negotiations with NSF, and annual advisory board meetings. Faculty experts served on advisory boards, one to advise our overall alliance model, and another focused on advising the social science research component of our alliance. Composition of the advisory boards was part of initial grant negotiations with the program officer to ensure a proper range of expertise among members in advising our alliance.

The alliance leadership team was not only open to receiving feedback, but actively sought it. An important development was establishing advisory boards to represent the cohort participants. Seeking feedback on behalf of the cohort participants regarding the content and direction of alliance activities became increasingly important over the lifecycle of the grant. This was in part due to increasing needs for customized and just in time support as cohort participants’ trajectories to the professoriate tended to diverge over time.

With so many sources of feedback, a systematic approach for responding to and incorporating recommended changes improves the potential for feedback to serve its purpose in improving alliance function. One approach in response to feedback from advisory board members was to organize the feedback in structured response documents that integrated the feedback and leadership team responses in a two-column format. This approach engaged leadership team members in dialog about how to address given feedback on a point-by-point basis.

The next step in the response cycle was for the leadership team to implement these responses or suggested solutions. An analysis of responses to advisory board feedback provided in the first two years of the program suggested that alliance members were least able to implement feedback in relation to improving model development, and better at creating collaboration and connection among alliance partners and cohort participants, connecting social science research to cohort members, and adopting specific dissemination plans for research.

During the virtual annual meeting held in June 2020, an advisory board member expressed interest in seeing how the leadership team incorporated previous feedback provided by the board. This comment revealed a gap in the feedback response cycle of illustrating the results of feedback, which brings the feedback process full circle to follow up in seeking continued feedback. Further, integrating and tracking feedback across sources and over time would provide a visible and coherent approach to engaging in meaningful cycles of inquiry, driving the improvement process.

### Researchers in Academia (R)

Program and evaluation stakeholders:

1. have a reciprocal relationship to the academic research base
2. use the research base to design project elements, best practices, measure outcomes
3. learn about and contribute findings to the research base:
  - AGEP sponsored conferences, webinars, and workshops
  - aligned conferences and communities of practice
  - publications in education, evaluation, and social science research journals

The introduction began with a discussion based on prior research of the benefits and burdens of collaborative practice. The academic research base is a product of prevailing scientific practice and the national and global intellectual climate regarding the value of collaborative practice. Stakeholders engage in reciprocal relationships with the research base on collaborative practice, both relying on it to inform their practice and shaping it with the results of that practice—the goal of the academic enterprise. For example, this current paper is a carefully considered contribution to collaboration research informed by the academic research base. For our alliance to progress beyond localized impacts of implemented activities, our leadership team must promote systematic engagement with the research community beyond our alliance. The dissemination of alliance findings to the larger academic community is an expectation for all AGEP alliances.

While the peer-reviewed literature is the gold standard of academic research, it is but one of a set of practices that can effectively disseminate findings and best practices to communities of interested researchers and practitioners. The following two sections are particularly important in the collaborative practice discussion, as they illustrate two structures with potential for widespread impact across the academic ecology. These are collaborative writing and dissemination practices across stakeholders as well as the engagement of our alliance stakeholders in a robust community of practice with the entire community of AGEP alliances.

#### Collaborative Dissemination

NSF program officers review alliance progress disseminating results from the development, implementation, and study of our AGEP alliance model as part of the annual reporting process. As part of grant negotiations with the program officer, the leadership team generated a detailed dissemination plan for education, evaluation, and social science research presentations and publications over the lifecycle of our funded alliance. The timeline itemized the title, research questions, first author, other authors, type of product (Conference, Journal, Instrument), and submission date.

While the need for dissemination support to fulfill the promised timeline was not evident during proposal development, it became so over the alliance lifecycle. Consider the requirements to successfully publish in peer review journals: both NSF and the partnering institutions require IRB approval for instrument selection, development, and acquisition procedures to collect data from human subjects. Limited capacity for consistent coordination of the alliance IRB application and amendments in the local team at the lead institution shifted the burden to the internal evaluator. This highlights the need to ensure the appropriate assignment of IRB responsibility and maintenance as part of the leadership team’s management responsibilities, particularly when management requires coordination across multiple university partners. Based on feedback from cohort participants, more careful coordination of data collection needs reduces the number of times cohort participants are asked to respond to queries and provides a comfortable time period in which to respond. Minimizing burden on cohort participants is particularly important in alliances with a large leadership team and requires considerable coordination.

A consequence of the evaluation team’s focus on collaborative practice was limited capacity to generate data for the education research included in each institution’s dissemination plan. The primary responsibility for instrument design, data collection, analysis, and dissemination of education research shifted to local institutional team members. The shift impacted some institutional teams more than others. A number of leadership team members were simply not familiar with research methods employed in education and social science research and required interdisciplinary collaboration with leadership team members who were. In particular, the evaluation team and faculty from the lead institution’s teaching center for excellence assisted institutional teams with dissemination products. The leadership team also sought input on dissemination strategies during advisory board meetings, conferences, and site visits.

To provide more direct support of stakeholders to achieve planned dissemination products, the evaluation team identified several strategies that successfully engaged alliance stakeholders in collaborative writing practices. These practices support scholarly productivity in team environments. A brief discussion of each collaborative practice follows, with each representing a different stakeholder team:

1. dissemination product teams
2. toolkit working group
3. synchronous writing circles

Dissemination product teams worked with a dissemination support consultant using virtual, shared, and synchronous spaces to prepare products to submit for publication and presentation. The consultant developed a needs assessment to review product status and initiate a work plan based on a collaborative approach to academic writing (Belcher, 2019). In the current context, the core structural practices to support dissemination teams are backward planning from identified product and submission requirements, writing with consistent focus on the argument the product is making, and working from a structured outline of the content using an accurately formatted draft document. The core behavioral strategies that build successful writing practices are the same as those that build successful habits: do not do it alone, do it daily, and do it in manageable pieces.

The toolkit working group assists leadership and institutional teams in sharing the alliance model with others in a public, accessible, durable, and virtual space. The toolkit development process employs a working group model organized around a charge. Shared interactive templates and drafted examples using accessible technology tools guided the structure and content of alliance activities and assisted institutional teams in gathering and presenting relevant details in the toolkit. The working group continues to draft the templates and examples with feedback from the leadership team during monthly meetings. This type of structure guarantees a consistent, accessible, and thorough description of alliance activities. The team is committed to employing technologies that are interactive and entice users to want to know more.

A final example engaged cohort participants in synchronous writing circles, a uniquely structured approach in which participants generated relevant academic content simultaneously during weekly virtual sessions lasting around one hour. During the spring of the fourth year, the lead faculty of the job search and preparation activity engaged cohort participants on specific job support activities such as research proposals or academic portfolios. A typical session would be to define the writing activity (write a specific aims section of a grant proposal) for 5–15 minutes, each circle participant works on their own writing for 30–45 minutes but remains active on the call or webinar, and each participant shares out about progress made for the final 5–15 minutes.

During the annual meeting held in July 2021, the activity team lead described the development and function of the circles. The cohort participants indicated their experience in these collaborative writing sessions as particularly helpful because being part of weekly sessions guaranteed hours of writing productivity on something relevant; each person was doing something similar but customized to their particular research interests. The common experience reinforced motivation and commitment, and the meeting structure helped create writing as a repeatable, accessible practice. A recent article in the Chronicle of Higher Education highlights a group that used a collaborative writing retreat to complete an edited collection volume about the origins of modern food habits.^12^ The most important lessons in these dissemination examples are recognizing that writing does not have to be solitary, and in fact should not be. Further, providing more direct support of planned dissemination products through dedicated personnel and collaborative writing practices are effective ways to increase scholarly productivity in alliance stakeholders.

#### AGEP Community of Practice

The mechanisms NSF program officers employed to engage the AGEP COP are worthy of emulation by other sponsoring programs desiring facilitated collaborative practice among a set of funded projects with similar goals. Our alliance took advantage of most if not all the AGEP COP offerings. NSF engages AGEP community members in a variety of activities including proposal review and site visit teams. Participating in these activities has been a valuable professional development opportunity for members of our alliance.

The AGEP program also supports conference hosting and attendance (e.g., the Boston AGEP National Research Conference (NRC);^13^ Boston University, 2021). Indeed, an NRC conference served as the catalyst for this paper and the others included in this special journal issue focused on diversifying the STEM professoriate (California Alliance, 2018). Our alliance has been an active participant in annual AGEP NRCs, sharing research and insights with the larger AGEP community. For example, our alliance shared an abbreviated version of our successful June 2020 annual alliance meeting with the AGEP COP during a workshop offered at the November 2020 NRC (Morris et al., 2020).

The AGEP program invested in building capacity in evaluation practices as demonstrated by their support of an Evaluation Capacity Building Conference^14^ (ECBC; Education Development Center (EDC), 2021) and building collaborative practice through their INCLUDES coordination hub^15^ (NSF, 2021b). The evaluation team started exploring the evolution of the SPARC model beyond the borders of our alliance to a focus on collaborative practice as reflected at the AGEP community level to develop shared conceptualizations and assessments of collaborative practice across alliances (Kelly et al., 2020b). They also sponsored a discussion at an ECBC webinar at the invitation of the team at EDC, engaging AGEP evaluators in a discussion and reflection about the evaluation of collaboration in their alliances (Kelly, 2021). Leveraging results across alliances allows stronger inferences about the impact of collaborative practice on stakeholders across the academic ecology and can build a shared understanding across the AGEP portfolio of 122 unique institutions of higher education. The critical point of these dissemination activities is to highlight the opportunities provided to work with the larger AGEP COP and how these opportunities enrich the research community dedicated to diversifying the professoriate.

### Community and Society at Large (C)

The entire academic ecology benefits when successful partnership projects:

1. support DEI in higher education and in the resulting STEM workforce
2. respond to contextual events in flexible and adaptable ways
3. expand knowledge, practices, and opportunities to benefit from collaborative practice in the partnership over time
4. encourage the transfer of best practices in collaborative practice to other partnerships and stakeholders to increase broader impacts
5. collaborate with other partnerships and stakeholders to expand research on collaborative practice generated by the AGEP COP

The larger academic and global (C)ommunity dictates the value of collaborative practice across stakeholders in the academic ecology. The value of collaborative practice is reflected in stakeholder perceptions of the positive impact of collaboration on project outcomes and by popular “demand” or adoption by others. In keeping with the focus of this special issue, the authors focus on the implications of the SPARC model for supporting DEI in higher education and pathways leading to diversification of the professoriate. The evaluation team of our alliance identified four collaborative practices that show promise for advancing DEI in higher education: advocacy roles for SPARC stakeholders, focus on well-being of academy scholars, virtual technologies to promote inclusive and equitable practices, and safe spaces for discussions about institutional racism and related topics.

The SPARC model emphasizes the role that AEPs can serve to facilitate the use of collaborative practice and its afforded benefits. It also emphasizes that all stakeholders in the AGEP ecology share responsibility for conceptualizing and actualizing collaborative practice. Stakeholders have power to influence their context—to use available avenues of expression to support the value of collaborative practice in service of DEI in the academy. Recent policy from AEA suggests that credible evaluation requires explicitly addressing DEI in the implementing context. In other words, evaluators are ethically obligated to advocate for social justice and cultural responsiveness in all evaluation activities.^16^

The pandemic provides a unique opportunity for research, as evidence continues to accumulate about how our thoughts, behaviors, leisure, work, and relationship with technology has changed. NSF issued a Dear Colleague Letter inviting the research community to think about critical research to capture during the pandemic period.^17^ Taking to heart the lessons learned during this unique time in history confirms the primary need to attend to the well-being of scholars from groups historically underrepresented in STEM fields in the academy, particularly in times of challenge. The medium of collaborative practice is one pathway to support well-being and academic success. Examples from our alliance were the virtual annual meeting and the monthly meetings of the SLC during the forced isolation period.

Due to the reliance on virtual meeting tools during the period of forced isolation, the evaluation team is studying the impact of technological tools used by alliances to promote inclusive and equitable practices in virtual spaces. The success of the annual meeting suggested that specific efforts to increase engagement through interactive tools can have positive results. Virtual technologies can also orchestrate interactions that ensure all participants engage in the content and provide feedback, an empowerment evaluation approach (Fetterman et al., 2017). A recent article suggested that remote learning can be used in similar ways to displace the roles of power and privilege that dominate the traditional classroom experience by decentralizing the teacher in learning, giving the power of engagement to the learners, increasing accessibility of information across multiple modalities, and employing equitable participation strategies to include everyone’s views.^18^ Both synchronous and asynchronous opportunities to view content across multiple modalities increases stakeholder access to information in ways most useful to them. The toolkit our alliance is developing will include a section that details interactive and inclusive technologies used by alliance stakeholders in providing alliance content to cohort participants or other alliance stakeholders.

The national dialog surrounding systemic racism and police brutality exploded upon the death of George Floyd by convicted felon Derek Chauvin. This incident, along with similar victims of police homicides, fueled the Black Lives Matter protest movement across the nation. Events such as the dispute at University of North Carolina in Chapel Hill over granting tenure to *1,619 Project* creator Nikole Hannah-Jones^19^ and recent legislation forbidding discussion of critical race theory in public schools^20^ further underline the urgency of our work to engage higher education in the challenge of achieving DEI across the academic ecology. As researchers concerned with DEI in the academy, it is critical to have forums to safely discuss these issues. From the perspective of the authors, the AGEP COP was not only a safe space in which to have an authentic dialog about these concerns, but also a community which considers this dialog an essential part of institutional change in higher education.

## Discussion

The goal of this method paper is to demonstrate the application of an evaluative model that spotlights collaborative practice across stakeholder groups in funded academic partnership programs. While this story reflects the perspective of our AGEP alliance, it mirrors the stories of other AGEP alliances. As such, it has relevance for the entire AGEP community and related STEM education partnership programs funded by NSF or other government sponsors.

The evaluation team summarized best practices and lessons learned for each SPARC stakeholder group into a reflection tool, the *SPARC Model Checklist for Collaborative Practice*. While targeted toward AEPs, other alliance stakeholders will find the checklist of value in their own collaborative practice. Given the goal of our alliance to promote diversification of the professoriate, the model highlights the benefits of collaborative practice in supporting stakeholders from groups historically underrepresented in STEM fields across outcome domains: partnership project implementation and performance, academic success and scholarly productivity, psychosocial adjustment, and physical and psychological well-being. The next section summarizes the content of the checklist. A full copy is available online.

### SPARC Model

#### (S)ponsor

Sponsor requirements for partner collaboration and program management drive what Partners consider when planning programs. When seeking funding support through a sponsored program, consider how expectations for collaborative practice are negotiated and communicated throughout the period of support.

- Does the solicitation include language about collaboration?
- During award negotiations:
  - Is there a focus on collaborative practice?
  - Does the sponsor require an evaluation team and advisory board?
  - Does the sponsor require separate applications from each institution?
- Is there a community of practice promoted by the sponsor?

#### (P)rogram

Program partners and participants must necessarily work together to propose, develop, implement, and study the alliance model for diversifying the professoriate. Consider how partners and participants incorporated collaborative practice in the procurement and execution of the alliance program.

- Was the program planned collaboratively?
  - Has the program team leveraged prior collaborations?
  - Did the program team engage evaluators and advisory boards during planning?
  - Did the program team engage grant writers to facilitate writing the proposal?
- Is the program implemented collaboratively?
  - Has the program team organized itself to respond effectively to disruptions?
  - Is technology intentionally incorporated to facilitate collaboration?
  - Are annual meetings or retreats planned intentionally to facilitate connection and collaboration?
- Have the participants in the program self-organized to collaborate?

#### (A)ssessment and Evaluation Professionals

Evaluators have a unique opportunity to promote collaborative practice by structuring evaluation explicitly around it. They can promote equity in collaboration to ensure equal representation of views. They can regularly spotlight collaborative practices they observe and support team dissemination activities (e.g., promoting sharing of data, collaborative tools, and studies across alliances). Based on the adoption of the CEIF (Woodland and Hutton, 2012) as a framework for evaluating collaborative practice within the alliance, the following questions align to the five entry points identified on the CEIF.

- Has your project team addressed how to operationalize collaborative practice?
  - Does the project team have a shared understanding of the program’s goals?
  - Has the project team created well-defined and documented structures and procedures for collaborative practice?
  - Has the project team provided communities of practice for cohort participants?
- Are you tracking participants’ and the team’s engagement in the program’s communities of practice?
  - How engaged are team members (i.e., number of members they engage with)?
  - Do team members’ connection patterns within and across institutions change over time?
  - Is there an evaluation team with internal, external, and advisory board components?
  - Is there a program director or coordinator who has primary responsibility for alliance management?
- Are you adjusting the content of your annual assessments to align with your program team’s status as they move through the stages of partnership development?
  - Forming: Are team members committed to a shared goal? Relationships established?
  - Norming: Have team members determined decision-making? Clarifying structures and processes?
  - Performing: Is the team focused on implementation? With minimal oversight?
  - Transforming: Is the team focused on dissemination and next steps for the partnership?
- Are you assessing the program team’s levels of integration?
  - Are levels of integration consistent across stakeholders? Across institutions?
  - Do levels of integration change over time? For which categories?
  - Do levels of integration reflect desired levels of sharing, co-hosting, or collaboration? Do they suggest any issues in need of attention?
- Are you assessing the program team’s cycles of inquiry?
  - Does the leadership team receive feedback from multiple sources (such as Advisory Boards, site visits, annual evaluation reports)?
  - Is the leadership team responsive to feedback in a concrete way?
  - Do team members share consistent opinions about how well their institutional team collaborates around data driven decision-making? How about the overall alliance team?

#### (R)esearchers

In the case of our AGEP alliance, we systemically contribute to the national conversation about the role of collaboration in partnership programs like AGEP through systematic dissemination. Based on our alliance work, consider the following questions about collaborative practice for alliances when thinking about research and dissemination.

- Does the program team use the academic research base to support their planning of collaborative practice?
- Does the program team have specific dissemination plans to share their alliance research or collaborative practice?
  - Do any team members require support for social science or education research?
  - Is there a team member with a clear responsibility for IRB coordination across institutions?
- Are team members engaging in group writing, coordinated workgroups, or other models of collaborative dissemination?
  - Would team members benefit from professional development in collaborative dissemination practices?
  - Would team members benefit from expert coaching or writing support?
- Do team members actively participate in the AGEP COP?
  - Do team members regularly share alliance work with the AGEP COP?
  - Have team members engaged in any collaborative work with other AGEP alliances?

#### (C)ommunity

The unprecedented health crisis and civil unrest of the past two years has forever altered the face of our national and global society. AGEP alliances occur within this context, and thus must remain responsive to the evolving conditions in which a program finds itself. Consider the following questions about being prepared for the future.

- Have alliance members considered how to promote the use of collaborative practice more broadly in diversifying the professoriate in their own role?
- Are there contingency plans in the case of disruptions to planned activities?
- Are mechanisms ensuring the well-being of all cohort participants in place?
- Are there safe spaces for the open discussion of concerns and solutions regarding DEI in the academy?

## Conclusion

The evaluation team of our AGEP alliance recognized the increasing value of collaborative practice in the design, implementation, evaluation, and dissemination of findings in the partnership over time. Authors operationalized the SPARC model with a checklist to assist program stakeholders in designing for and assessing collaborative practice in support of project goals in funded academic partnership projects, emphasizing the contributions of collaborative practice in promoting diversification of the professoriate.

Before concluding, a word from our authors. During our work on the AGEP alliance, we were cognizant of the transformational contributions of this moment in time and reflective practice in creating the SPARC model of collaboration. The combination of a unique point in history with being intentional about learning from the experience created a mindfulness that guided us to important insights about collaborative practice. Engaging in reflective practice helps your brain make sense of the value of something to you and how you will use it (Bransford et al., 2000). Thus, our most important advice in conclusion is to be mindful and observant of the role of collaborative practice and how to structure it in a way that offers value to all group members.

Both a strength and a limitation of the SPARC model presented here is its “*post hoc*” rather than *a-priori* design, an emergent phenomenon that compelled our attention as we hope it will compel yours. As such, it should be considered an initial model, based on a strong yet small set of data. As evaluators, we evolved our approach to collaborative practice and its assessment over the years of the alliance, and this will certainly continue in the last year of the program. Regardless of its rigor in this initial form, it does provoke a rich discussion about collaborative practice that can have immense value for enhancing programs that promote diversification of the professoriate.

An additional limitation is the extent of the body of work reviewed here. The work presented is based on annual interviewing of around 30 people per year, in addition to attendance and observations of meetings and professional development that require considerable time for data collection and analysis. Further, evaluators must have the capacity to conduct the qualitative research and data analysis described. Application of the framework requires flexibility of the evaluator to design evaluation questions based on the collaboration development stages. The authors have decades of combined experience in evaluation work of this nature. Also, alliance members or grant recipients must be willing to invest time to participate in these interviews and be comfortable sharing their views about the project with the evaluators.

A current focus of the evaluation team is exploring ways to leverage common results across alliances. When attending NRC conferences, it is quite common to hear a presenter echo something the evaluation team has observed in our alliance. If there were systematic efforts to build on these common findings, the work of the AGEP COP could take a new direction. In the results, we discussed initial work to create a way to characterize collaborative practice across alliances for the sake of comparing alliance practices more directly (Kelly et al., 2020b); foundations already exist for this future work.

The results outline an emergent model of collaborative practice across key stakeholder groups in the academic ecology of a funded alliance. This alliance is part of the AGEP program in NSF, a sponsored program focused on increasing the diversity of the professoriate. The SPARC model encourages a broader conceptualization of the potential benefits of collaborative practice because it extends beyond alliance boundaries and demonstrates what each stakeholder group uniquely contributes to collaborative practice in the academic ecology. Collaborative practice is a key transdisciplinary skill set (Kelly and Burr, 2019), worthy of substantial investment.

“The ability to collaborate on both a large and small scale is one of the core requisites of post-modern society … in short, without collaborative skills and relationships it is not possible to learn and to continue to learn as much as you need in order to be an agent for social improvement.” (Fullan, 1994, pp. 17–18).

## Data Availability Statement

Due to confidentiality concerns and limitations of sample size, the datasets presented in this article are not publicly available. Requests for data or further details should be directed to Theresa Murphrey, t-murphrey@tamu.edu.

## Ethics Statement

The studies involving human participants were reviewed and approved by Texas A&M University TAMU 1186 | College Station, TX 77843 Tel. 979.458.4067 | Fax. 979-862-3176, https://vpr.tamu.edu/human-research-protection-program/. Written informed consent for participation was not required for this study in accordance with the national legislation and the institutional requirements. Signed informed consent from all program stakeholders (both those receiving programming and those delivering programming) allowed the use of ongoing implementation data collected as part of the project for research and evaluation purposes. Specific instruments were also submitted for approval, including protocols and instruments used in evaluating collaborative practice.

## Author Contributions

EB, KK: method conceptualization. EB, KK, TM, and TK: instrument development. EB, KK, and TM: data collection. EB, KK, TM, and TK: analysis and interpretation of results. EB, KK, TM, and TK: draft manuscript preparation. All authors contributed to the article and approved the submitted version.

## Funding

This work was supported by the National Science Foundation Alliances for Graduate Education and the Professoriate (AGEP; solicitation NSF 16-552) under award numbers 1723255, 1723260, 1723165, and 1723253. It was also supported by Oak Ridge Associated Universities under the 2020 Thought Leadership Research Awards (TLRA) program. Any opinions, findings, and conclusions or recommendations expressed in this article are those of the author(s) and do not necessarily reflect the views of the National Science Foundation or Oak Ridge Associated Universities.

## Conflict of Interest

The authors declare that the research was conducted in the absence of any commercial or financial relationships that could be construed as a potential conflict of interest.

## Publisher’s Note

All claims expressed in this article are solely those of the authors and do not necessarily represent those of their affiliated organizations, or those of the publisher, the editors and the reviewers. Any product that may be evaluated in this article, or claim that may be made by its manufacturer, is not guaranteed or endorsed by the publisher.

## Footnotes

^1^ https://www.nsf.gov/about/congress/reports/nsf_big_ideas.pdf

^2^African Americans, Hispanic Americans, American Indians, Alaska Natives, Native Hawaiians, and Native Pacific Islanders.

^3^ https://www.nsf.gov/funding/pgm_summ.jsp?pims_id=5474

^4^SUNY at Stony Brook, Texas A&M University, Tuskegee University, University of California-Berkeley, and University of Maryland Baltimore County led multiple AGEP alliance projects.

^5^ https://socnetv.org/

^6^ https://about.citiprogram.org/en/homepage/

^7^ https://www.eval.org/

^8^ https://comm.eval.org/stemeducationandtraining/home

^9^ https://beta.nsf.gov/funding/opportunities/national-science-foundation-research-traineeship-program

^10^ https://www.nordp.org/resource-links

^11^ https://www.research.gov/research-web/content/aboutprojectreports

^12^ https://www.chronicle.com/article/lessons-for-academics-from-a-weekend-writing-retreat?cid2=gen_login_refresh&cid=gen_sign_in

^13^ https://live.bu.edu/agep/

^14^ https://agep-ecbc.edc.org/

^15^ https://www.includesnetwork.org/home

^16^ https://www.eval.org/About/About-AEA/Mission-Vision-Values

^17^ https://www.nsf.gov/pubs/2020/nsf20052/nsf20052.jsp

^18^ https://www.edsurge.com/news/2021-09-06-how-remote-learning-subverts-power-and-privilege-in-higher-education

^19^ https://www.npr.org/2021/06/30/1011880598/after-contentious-debate-unc-grants-tenure-to-nikole-hannah-jones

^20^ https://www.brookings.edu/blog/fixgov/2021/07/02/why-are-states-banning-critical-race-theory/
